# Testing a Firefly-Inspired Synchronization Algorithm in a Complex Wireless Sensor Network

**DOI:** 10.3390/s17030544

**Published:** 2017-03-08

**Authors:** Chuangbo Hao, Ping Song, Cheng Yang, Xiongjun Liu

**Affiliations:** Key Laboratory of Biomimetic Robots and Systems (Ministry of Education), Beijing Institute of Technology, Beijing 100081, China; 3120130073@bit.edu.cn (C.H.); ycthink@gmail.com (C.Y.); 3120140085@bit.edu.cn (X.L.)

**Keywords:** wireless sensor networks, firefly-inspired algorithm, coupling strength, multiscale model, stochastic coupling

## Abstract

Data acquisition is the foundation of soft sensor and data fusion. Distributed data acquisition and its synchronization are the important technologies to ensure the accuracy of soft sensors. As a research topic in bionic science, the firefly-inspired algorithm has attracted widespread attention as a new synchronization method. Aiming at reducing the design difficulty of firefly-inspired synchronization algorithms for Wireless Sensor Networks (WSNs) with complex topologies, this paper presents a firefly-inspired synchronization algorithm based on a multiscale discrete phase model that can optimize the performance tradeoff between the network scalability and synchronization capability in a complex wireless sensor network. The synchronization process can be regarded as a Markov state transition, which ensures the stability of this algorithm. Compared with the Miroll and Steven model and Reachback Firefly Algorithm, the proposed algorithm obtains better stability and performance. Finally, its practicality has been experimentally confirmed using 30 nodes in a real multi-hop topology with low quality links.

## 1. Introduction

Data acquisition is the foundation of soft sensor and data fusion. In industrial processes, data often comes from multiple sources, which may be separated physically or virtually, and need to be synchronized. Therefore, distributed data acquisition and its synchronization are the important technologies to ensure the accuracy of soft sensors [1,2]. At present, Wireless Sensor Networks (WSNs), as an important distributed data acquisition technology, have been widely used in industry [3,4,5]. Synchronization is a key feature of WSNs, and it has been used in many wireless protocols. In general, the synchronization algorithm can be classified according to the centrality. On one hand, the centralized algorithm (e.g., Reference Broadcasts Synchronization [6], Timing-sync Protocol [7], and Flooding Time Synchronization Protocol [8]) sets a base time for the coordinator and the nodes modify their time correspondingly, thus the accuracy of this algorithm is high for structures with simple topology [9]. However, if the topological structure is complex, the centrality algorithm is inapplicable. On the other hand, compared with the centralized algorithm for synchronization, the distributed algorithm, which avoids single points of failure and is more robust, is more suitable for large-scale complex topological structures [10].

The distributed algorithm can be implemented with the classical packet-coupling synchronization or the firefly-inspired synchronization. The packet-coupling synchronization has accuracies ranging from microseconds to milliseconds [11]. However, a large number of packets need to be exchanged, which leads to increased computational complexity, energy expenditure, and poor scalability. Development of synchronization technology has led to the firefly-inspired synchronization [12,13,14]. It can obviate the drawbacks of packet-coupling methods emerging from pulse coupling. To date, most studies have relied on simulating firefly-inspired synchronization [15,16,17,18,19], because it is a physical layer-based method and depends heavily on the network hardware. Because firefly-inspired synchronization is not compatible with most commercially available WSN chips [20], very few groups have used firefly-inspired synchronization. Some groups have adopted firefly-inspired synchronization on special platforms such as a Wire [21,22], Ultra Wideband (UWB) [23], Custom Radio [24] and Light Emitting Diode (LED) [25].

To implement the firefly-inspired synchronization algorithm on more hardware platforms, this algorithm has recently been transformed into a software algorithm on a low Media Access Control (MAC) layer combined with packet-coupling synchronization [11], and is called the Reachback Firefly Algorithm (RFA). Based on the Miroll and Steven model (M&S model) [26], Werner-Allen et al. first utilized RFA with realistic radio effects and theoretically confirmed its stability [20]. The algorithm was tested on a 24-node indoor WSN and it was found that 50% of the nodes achieved synchronization within 154 μs [20]. However, the selection of the coupling strength (also called coupling factor, the definition can be found in [27]) and a random avoid time (the random transmission delay to node firing messages for avoiding repeated collisions) were not considered, which may influence the stability. Rather, the algorithm was simulated within a defined coupling strength range. Leidenfrost improved the theory of RFA and suggested the scope of avoid time and coupling strength in an all-to-all network where each node can communicate with every other node. However, in a complex topology, the time spread only reached milliseconds [27]. The refractory period is often assigned as a duration of time right after firing, during which no signal can be received from other nodes. Cui found that it had a positive effect on the convergence in RFA, however the design of the coupling strength was not taken into consideration [28]. A biologically inspired approach for distributed slot synchronization similar to RFA was implemented on a MAC layer and achieved slot synchrony in slotted communication systems by Tyrrell [29]. However, the synchronization was not tested on a realistic WSN and the design of the coupling strength was not considered. The simulation results showed that a coupling strength that was too high or low would lead to instability of the synchronization. Therefore, the coupling strength is still an open question for WSNs with complex topology, and it is difficult to select an appropriate coupling strength in a complex or a changeable topology. This problem is regarded as one of the most significant in the field of firefly-inspired algorithms [30]. The conflicts between synchronization messages have been present since synchronization was first developed [20,27]. Some researchers use a random avoid slot time to ease the conflicts, and the range of the avoid slot may affect the stability [20,27]. However, the range of the avoid slot was presented only for an all-to-all network, which is not suitable for a realistic WSN [27]. Hence, the conflicts between synchronization messages in a high-density network are still unresolved.

The focus of this paper is reducing the design difficulty of the firefly-inspired synchronization algorithm and to quantify the performance tradeoff between network scalability and synchronization capability in complex WSNs. We develop the discrete multiscale phase dynamics and design a stochastic coupling algorithm. The dynamics transform the phase into a state vector according to different quantitative levels. A node in the WSN broadcasts its state vector stochastically, which is then adjusted after receiving a neighboring state vector from a low MAC layer. The phase adjustment happens on each quantitative level with a constant adjustable step size, which not only ensures stability according to the global convergence property of Markov chains [31] without changing the coupling strength, but also achieves a more accurate and faster synchronization. In this paper, frequency drift and communication delay are also considered. The stability of the proposed algorithm is proven theoretically, and confirmed by comparing simulations with RFA in a complex WSN. It is found that the proposed algorithm achieves better performance. Finally, the algorithm is tested in a realistic, complex WSN with 30 nodes and achieves synchronization within 50 μs. Compared with the M&S model and RFA, this method has the following advantages:

1. By using a discrete state vector instead of a continuous phase, the algorithm can be easily implemented on a Microcontroller Unit (MCU) in a wireless module.
2. The step size can be adjusted, which ensures the stability according to the global convergence property of Markov chains without adjusting the coupling strength.
3. A better performance emerges from the stochastic coupling at multiscale quantitative levels and the channel congestion is alleviated significantly.

The rest of this paper is organized as follows: in Section 2, the discrete phase dynamics at multiscale quantitative levels is proposed. Section 3 presents the design of a stochastic coupling algorithm, including the compensation for frequency drift and communication delay. The stability of this algorithm is verified in Section 4. Simulation and experiment results are discussed in Section 5 and Section 6. Finally, conclusions and future research directions are presented in Section 7.

## 2. Discrete Phase Dynamics

In this section, we generalize the integrating and coupling dynamics. According to the M&S phase model [26], the phase is discretized at a single-scale quantitative level, making it is easier to implement on a WSN node. The single-scale quantitative level is expanded to multiscale quantitative levels for better performance.

### 2.1. Discretization of a Single-Scale Quantitative Level

In the discrete phase, each node is characterized by a cycle-count timer *k* (the period is *T*). Assume that Δ*t* is a short duration of time which denotes the resolution of timer and *T* can be divided exactly by Δ*t*. The timer *k* increases by one for each Δ*t*. Thus, in each node, the local timer *k* can be expressed as a discrete phase, which consists of a series of moments *t* and:

(1) $$t\in \left\{k\Delta t|k\in Z,0\leq k<\frac{T}{\Delta t}\right\}$$

where *Z* denotes the set of integers, rather than the continuous phase contained in the M&S model. The dynamics of each node are composed of the integrating and coupling dynamics.

#### 2.1.1. Integrating Dynamics

The integrating dynamics are determined by the properties of the node, which does not consider the network coupling. The node’s phase would not be influenced by other nodes in integrating dynamics. The integrating dynamics represent the process of increasing the discrete phase *k* independently in each node as a counter, shown in Equation (2):

(2) $$t^{n+1}-t^{n}=\Delta t\Rightarrow \{\begin{array}{c} k(t^{n})=\frac{T}{\Delta t}-1\Rightarrow k(t^{n+1})=0\\ k(t^{n})\neq \frac{T}{\Delta t}-1\Rightarrow k(t^{n+1})=k(t^{n})+1 \end{array}$$

As time increases from *t^n^* to *t^n^*^+1^ with Δ*t*, the phase *k* becomes *k* + 1. When the phase reaches its phase threshold T/Δ*t*, *k*(*t^n^*^+1^) will return to zero immediately. From Equation (2), the period of the phase is *T* and the update interval is Δ*t* (which denotes the quantization resolution). Here, the quantitative level is defined as the threshold of the timer, as shown in Equation (3):

(3) $$k_{\max}=\frac{T}{\Delta t}-1$$

The timer *k* may be any natural number less than the quantitative level, and *T* is the threshold time corresponding to the phase threshold.

#### 2.1.2. Coupling Dynamics

The coupling dynamics adjust the phase *k* of a node coupled with another node in the WSN. For the purposes of the following description, suppose that node *j* receives the synchronization message of node *i*. _*i*−*j*_*Diff*(*_i_k*(*t*), *_j_k*(*t*)) represents the phase difference between *i* and *j* at moment *t*, where the phase in *i* and *j* is *_i_k*(*t*) and *_j_k*(*t*), respectively. The phase difference can be calculated from Equation (4), and *k* can be obtained by processing the synchronization packet. A further explanation is provided in Section 3.2 and Section 3.4.

(4) Diffi−j(ki(t),kj(t))={ki(t)−kj(t)+TΔt, when ki(t)−kj(t)<−T2Δtki(t)−kj(t),when −T2Δt≤ki(t)−kj(t)≤T2Δtki(t)−kj(t)−TΔt ,when T2Δt<ki(t)−kj(t)

From Equation (4), *_i_*_−*j*_*Diff* is an integer and meets −*T*/2Δ*t* ≤ *_i_*_−*j*_*Diff* ≤ *T*/2Δ*t_._* Considering the discretization, the phase is non-derivable and the adjustment of the phase must be divided exactly by Δ*t*. The adjustment of the discrete phase is determined by a step-increasing function for the discrete phase difference in order to retain the concave property of the phase mapping function [26]. In order to avoid cumbersome floating-point operations, the adjustment is determined according to a step-increasing function *f*(*_i_*_−*j*_*Diff*) as shown in Equation (5):

(5) f(Diffi−j)={1, when T2Δt−r>Diffi−j>r0, when |Diffi−j|≤r−1, when −r>Diffi−j>r−T2Δt

In Equation (5), *r* denotes the refractory period which usually meets *r* < 0.4*T*/Δ*t* [28]. Here, the refractory period is shown in terms of the phase difference. From Equation (5), the coupling dynamics transform the continuous phase adjustment into a phase state transition, similar to the state transition in a Markov chain. The details are shown in Section 4.1. The conversion conditions are shown in Equation (5), which are key to achieving synchronization. The stability is ensured by the property of Markov chains, which will be explained in Section 4. Finally, the coupling dynamics are presented in Equation (6):

(6) Node j coupling with Node i⇒∀j≠i,kj(t+)=kj(t)+f(Diffi−j)

**Figure 1:**
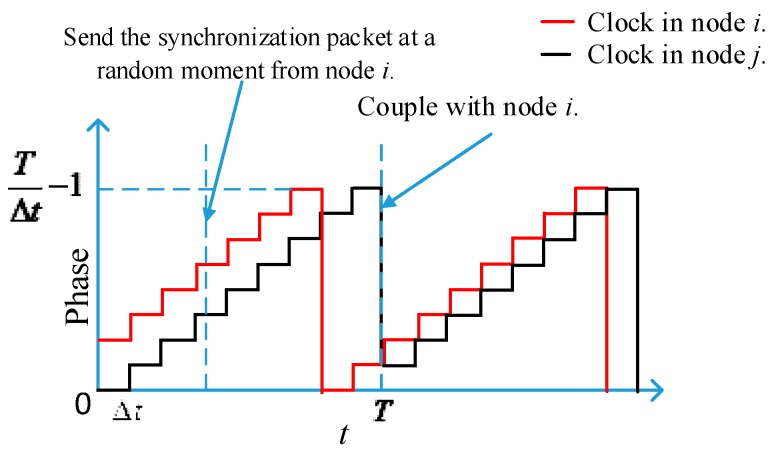
An example of coupling dynamics.

Figure 1 shows an example. In this figure, *_i_*_−*j*_*Diff*(*_i_k*(0),*_j_k*(0)) = 2 while the refractory period is 1, and node *j* is coupling with node *i*. Thus, *f*(*_i_*_−*j*_*Diff* ) = 1. The coupling occurs when the node *j* reaches its phase coupling (*t* = *T*). These details will be explained in Section 3.

### 2.2. Discretization of Multiscale Quantitative Levels

Discretization divides the period time into pieces. Quantitative level denotes the amount of pieces. According to [32], a larger quantitative level will lead to a higher synchronization accuracy. However, a large quantitative level will lead to a smaller Δ*t* and a larger number of state elements, which may lead to partial synchronization and slow the convergence. Therefore, multiscale quantization is adopted in this section. Utilizing multiscale quantitative levels, the synchronization process is accelerated by using large quantitative levels, and the accuracy is improved by using small quantitative levels.

Consider the quantization process with different levels. Each level has a different quantization resolution. After quantization, the discrete phase value is converted to a multiscale phase space. In order to decouple each level, we sort them hierarchically by quantitative level and define the threshold time *T* (*T* can be modified as needed) in one layer as the quantization resolution in next lower layer, as shown in Equations (7) and (8):

(7) $$k_{l\max}\Delta t_{l}=\Delta t_{l-1},T_{l}=\Delta t_{l-1}$$

(8) $$k_{l\max}=\frac{T_{l}}{\Delta t_{l}}(l\in \{0<l\leq m|l\in Z^{+}\})$$

where *l* denotes the layer index of quantitative level; *m* is the amount of layers and *k_lmax_* represents the quantitative level of layer *l*. Thus, when layer *l* is adjusted, the phase in other layers will not be affected unless the threshold is reached. The value of period and quantization resolution in each layer can be selected as needed.

The smallest Δ*t* determines the time resolution of the node, which is usually valued according to the accuracy requirement and hardware limitations. A smaller Δ*t* may get a higher time resolution and accuracy but require a more tedious calculation. Here we use the smallest Δ*t* to form a resolution vector ***RES***, as shown in Equation (9):

(9) $$\begin{array}{l} RES_{m}=1,RES_{l}=\prod_{n=l+1}^{m}k_{n\;\max}\\ \mathrm{RES}=\left[\begin{array}{c} RES_{1}\\ \vdots \\ RES_{m} \end{array}\right] \end{array}$$

Because the time resolution at the highest layer of quantitative level (the *m*th layer) is the smallest Δ*t*. Thus *RES_m_* = 1. In multiscale dynamics, the real phase value can be expressed by the discrete phase value in each layer. Combining the different phase values of *m* layers forms the state vector *_i_**K***(*t*) of the node *i*, as shown in Equation (10):

(10) Ki(t)=[k1i(t)k2i(t)⋮kmi(t)]

where *_i_k_l_*(*t*) represents the phase in *i* at the *l*th layer at the moment *t*.

The real normalized phase in node *i* can be rewritten as *_i_**K*** in Equation (11):

(11) Pih=∑l=1mkli⋅ΔtlT1max=KTi⋅RES⋅ΔtmT1max

In each layer, the integrating and coupling dynamics are the same as the dynamics of the single-scale quantitative level. According to Equation (2), the integrating dynamics at the *l*th layer are shown in Equation (12).

(12) $$t^{n+1}-t^{n}=\Delta t_{l}\Rightarrow \{\begin{array}{c} k_{l}(t^{n})=\frac{T_{l}}{\Delta t_{l}}-1\Rightarrow k_{l}(t^{n+1})=0\\ k_{l}(t^{n})\neq \frac{T_{l}}{\Delta t_{l}}-1\Rightarrow k_{l}(t^{n+1})=k_{l}(t^{n})+1 \end{array}$$

As with the single-scale dynamics, it is necessary to define the multiscale phase differences in a matrix before determining the multiscale coupling dynamics, shown in Equation (14):

(13) Diffli−j=Diffi−j(kli(t),klj(t))

(14) Diffi−j(Ki,Kj)=[Diff1i−jDiff2i−j⋮Diffmi−j]

where *_i_*_−*j*_*Diff_l_* denotes the phase difference at the *l*th layer of quantitative levels between node *i* and node *j*.

The step-increasing function can be determined by mapping the phase difference matrix into the adjustment step vector as shown in Equation (15):

(15) F(Diffi−j(Ki,Kj))=[f(Diff1i−j)f(Diff2i−j)⋮f(Diffmi−j)]

where *f* can be found by Equation (5). Thus, the coupling dynamics at multiscale quantitative levels are shown in Equation (16):

(16) Node j coupling with Node i⇒∀j≠i,Kj(t+)=K(t)+F(Diffi−j)

## 3. Stochastic Coupling Synchronization Algorithm

**Figure 2:**
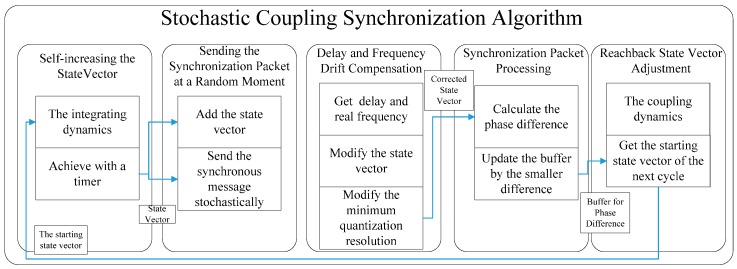
The block diagram of the stochastic coupling synchronization algorithm.

Based on the dynamics presented in Section 2, a stochastic coupling synchronization algorithm is proposed in this section, as shown in Figure 2. It consists of five processing tasks:

1. Self-increasing the state vector.
2. Sending the synchronization packet at a random moment.
3. Delay and frequency drift compensation.
4. Synchronization packet processing.
5. Reachback state vector adjustment.

### 3.1. Self-Increasing the State Vector

According to the integrating dynamics, the state vector should be updated to meet Equation (12). Unlike the general continuous phase model, the phase growth in each layer is step-like. As described in Section 2, the discrete phase value should increase by one after time Δ*t_l_* in the *l*th layer. Thus, the state vector should be updated at each time point Δ*t_m_*, which is easy to achieve with a timer in the MCU.

### 3.2. Sending the Synchronization Packet at a Random Moment

**Figure 3:**
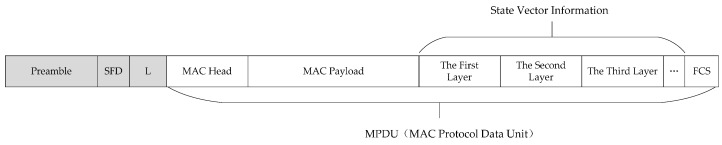
Synchronous message structure.

The algorithm adopts a stochastic coupling method. The triggering synchronization packet includes the regular data format of the wireless network and the state vector information is added at the end of the MAC Payload. It is compatible with existing mainstream network formats (e.g., IEEE802.11 and IEEE802.15.4). Figure 3 illustrates the synchronous message structure of IEEE802.15.4 as an example. The phase information in this figure presents the state vector of the trigger node, and the coupling can be realized in each layer. Unlike the traditional triggering method, the synchronous message sending is carried out stochastically during the synchronization. In addition, a synchronous message can be combined with other network transmission packets to reduce the network load. In addition, we employ the Carrier Sense Multiple Access/Collision Avoidance (CSMA/CA) for the idle listening which is compatible with IEEE802.11 or IEEE802.15.4. The Maximum Transmission Unit (MTU) depends on the protocol and the application communication requirements.

### 3.3. Delay and Frequency Drift Compensation

Due to the communication delay and in order to reduce the instability of the algorithm, it is necessary to compensate by optimizing and calibrating the delay. The total communication delay *t*_dly_ satisfies the relationship in Equation (17):

(17) $$t_{\mathrm{dly}}=t_{\mathrm{uc}}+t_{c}\approx t_{c}$$

where *t*_uc_ denotes the uncertain time delay, which includes the sending time, access time, and receiving time. The send time is that of the sender constructing the time message to transmit on the network. The access time is that of the MAC layer delay in accessing the network. Finally, the receive time is the receiving node processing the message and transferring it to the host. *t*_uc_ can be nearly eliminated with the Start Frame Delimiter (SFD) timestamp technique [28]. In IEEE802.11, chips may not provide the SFD interrupt nor the SFD output pin. Instead, Transmit (TX) start interrupt and Receive (RX) end interrupt can be utilized. Then calculate the timestamp by the TX or RX timing. *t*_c_ denotes the deterministic delay, which is the difference between the running and response times, which is caused by the interrupted service that is a function of sending and receiving SFD, and can be obtained by calibration. After obtaining the communication delay, it is necessary to convert the delay into the form of a state vector. The conversion method is shown in Equations (18) and (19):

(18) $$k_{\mathrm{dly},l}=(t_{\mathrm{dly}}-\sum_{n=l+1}^{m}k_{\mathrm{dly},n}\Delta t_{n})\;\mathrm{mod}\;\Delta t_{l}$$

(19) $$K_{\mathrm{dly}}=\left[\begin{array}{c} k_{\mathrm{dly},1}\\ \vdots \\ k_{\mathrm{dly},m} \end{array}\right]$$

Since the deterministic delay can be calculated in advance, the state vector of the delay can be calculated in advance, and there is no need to calculate it again while the process is running. The modified state vector meets the relationship in Equation (20). Thus, a more accurate state vector ***K*** can be obtained:

(20) $$K\prime (t)=K(t)-K_{\mathrm{dly}}$$

A low-performance RC oscillator is often used as the clock driver in WSNs for cost savings, which introduces a large frequency drift error. To reduce this error and improve the synchronization accuracy, this algorithm employs the correction algorithm presented in [25]. First, the real frequency *f*_RC_ of the RC oscillator is calibrated and compared with the theoretical frequency *f*_ideal_. The adjusted minimum quantization resolution is obtained on the basis of the theoretical minimum quantization resolution Δ*t_m_*, shown in Equation (21):

(21) $$\Delta t_{1}\prime \;=\;\frac{\Delta t_{1}f_{\mathrm{RC}}}{f_{\mathrm{ideal}}}$$

### 3.4. Synchronization Packet Processing

In the synchronization process, the node receives the synchronization packet sent by the neighboring node and couples with each layer. During the processing, there is a buffer that only stores the difference of the state vector, which is the smallest in a period of the refractory interval. If we assume that node *j* has received a synchronization packet sent by node *i*, then the process is as follows:

1. Node *j* should record the local state vector *_j_**K*** exactly when receiving the synchronization packet of node *i* and get the corrected state vector by compensation as described in Section 3.3.
2. Analyze the synchronization packet to obtain node *i*’s state vector *_i_**K***.
3. According to Equation (14) calculate the difference *_i_*_−*j*_***Diff***(*i**K***,*j**K***). Due to the discretization of phase, when the phase difference equal to 1 in a certain quantitative layer, the real continuous difference value should be less than the time resolution in this layer. So the difference should be reflected in the former layer which has a more precise time resolution. Thus, in order to prevent an oscillatory response between two adjacent layers when the synchronization is almost achieved, it should be equivalently converted to the former layer as shown in Equation (22):
  (22) Diffi−j(kl+1i(t),kl+1j(t))=±1⇒{Diffi−j(kl+1i(t+),kl+1j(t+))=0Diffi−j(kli(t+),klj(t+))=Diffi−j(kli(t),klj(t))±klmax
4. If *_i_*_−*j*_***Diff***(*i**K***,*j**K***) in Step 3 is out of the refractory interval, update the buffer *_i_*_−*j*_***Diff***_buf_ by the smaller difference between the one just obtained and the one stored in the buffer. Thus, after a period, the buffer only stores the difference of state vector that is the smallest in the period. That is to satisfy Equation (23):
  (23) |Diffbufi−j(t)T⋅RES|>|Diffi−j(Ki,Kj)T⋅RES|and|Diffi−j(Ki,Kj)T⋅RES|≥r⇒Diffbufi−j(t+)=Diffi−j(Ki,Kj)|Diffbufi−j(t)T⋅RES|≤|Diffi−j(Ki,Kj)T⋅RES|or|Diffi−j(Ki,Kj)T⋅RES| ≤r⇒Diffbufi−j(t+)=Diffbufi−j(t)
  where ***RES*** is the resolution vector in Equation (8) and *r* is the length of the refractory interval in [28]. The phase difference in the buffer is used to provide input for the reachback state vector adjustment.

### 3.5. Reachback State Vector Adjustment

As the node’s hardware constraints and receiving and adjusting the phase right after receiving the packet may cause hysteresis and instability, the adjustment uses the reachback mechanism of RFA [20]. Under that mechanism, after the longest time threshold, the node obtains the state vector difference in the buffer and calculates ***F***(*_i_*_−*j*_***Diff***_buf_) as the starting state vector of the next cycle, shown in Equation (24). This can reduce the amount of computing and avoid the impact of disordering reception:

(24) km=kmmax⇒K=F(Diffbufi−j)

## 4. Stability

As the adjustment in one layer does not affect the other layers, the stability of the target algorithm can be determined by verifying the stability in a single layer. Therefore, this section focuses on proving the stability of the model with a single-scale quantitative level. The stability of the two-node network is demonstrated, and the conclusions are extended to a multi-node network.

### 4.1. Stability of a Two-Node Network

#### 4.1.1. State Space of the System

As shown in Section 3, all possible values of the discrete phase in node *i* can be gathered in a set as shown in Equation (25):

(25) Xi={ϕki=kΔϕ,k∈D}D={n∈Z|0≤n≤1Δϕ}

To explain this process clearly, suppose *T* is an even multiple of Δ*t*. Therefore, *_i_**X*** is a finite state space of the node itself and each node can be regarded as a finite state machine (FSM), which constitutes a Markov chain.

Similarly, for a two-node network, the absolute value of *_i_*_−*j*_*Diff* can be selected as the state of the network. All possible values can be gathered in a set as shown in Equation (26):

(26) |Diff|i−j={|Diff|(k)i−j=kΔt,k∈D}D={k∈Z|0≤k≤T2Δt}

Hence, the system state can be modeled by a discrete-time Markov chain. Synchronization is achieved when the discrete phase difference between the two nodes decreases to zero.

#### 4.1.2. One-Step Transition Probability Matrix

For nodes *i* and *j*, suppose they satisfy *_i_k* > *_j_k* and:

(27) ki−kj=Diff(ki−kj)i−j while Diff(ki−kj)i−j≤T2Δt

Consider the next adjustment of the phase in a two-node network. From Equation (5), we can see that the adjustment step is −*f*(*_i_*_−*j*_*Diff*), which always has an opposite sign of the input *_i_*_−*j*_*Diff*. Hence, the adjustment is a negative feedback and always reduces the absolute value of *_i_*_−*j*_*Diff*.

Therefore, if the synchronization has not yet been achieved, the probability of state transition from the adjustment *l* to (*l* + 1) can be found from Equations (28) and (29):

(28) P(|Diff|(ki−k−1j)l+1i−j||Diff|(ki−kj)li−j)=1

(29) P(|Diff|(ki−kj)l+1i−j||Diff|(k)li−j)=0 when k≠ki−kj−1,Diff(k)li−j∈Diffi−j

**Figure 4:**
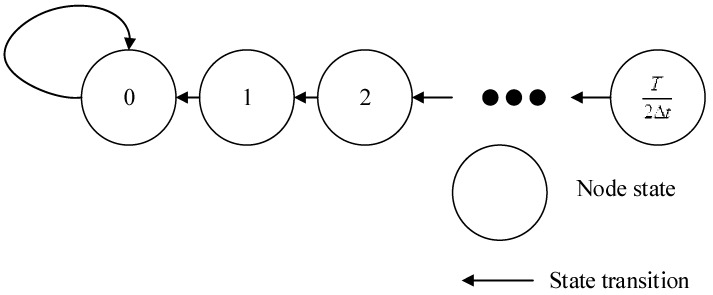
The Markov chain of the two-node system.

For the two-node system that has a state space shown in Equation (30) and the Markov chain is shown as Figure 4:

(30) $${\left[\begin{array}{lllllll} 0 & \cdots & n-1 & n & n+1 & \cdots & \frac{T}{2\Delta t} \end{array}\right]}^{T}$$

The one-step transition probability matrix is constructed as shown in Equation (31):

(31) $$P=\left[\begin{array}{cccccccc} 1 & \cdots & 0 & 0 & 0 & \cdots & 0 & 0\\ \vdots & \ddots & \vdots & \vdots & \vdots & & \vdots & \vdots \\ 0 & \cdots & 0 & 0 & 0 & \cdots & 0 & 0\\ 0 & \cdots & 1 & 0 & 0 & \cdots & 0 & 0\\ 0 & \cdots & 0 & 1 & 0 & \cdots & 0 & 0\\ \vdots & & \vdots & \vdots & \vdots & \ddots & \vdots & \vdots \\ 0 & \cdots & 0 & 0 & 0 & \cdots & 0 & 0\\ 0 & \cdots & 0 & 0 & 0 & \cdots & 1 & 0 \end{array}\right]$$

#### 4.1.3. Proof of Stability

Suppose the state is *n* (*n* ≥ 1). After one step transition, the state will be *n* − 1 according to the Equation (31). And if the state is 0, after one step transition, the state will always be 0. Since the max of state is *T*/2Δ*t* in Equation (30), after *T*/2Δ*t* steps, the state will be 0 constantly and the probability of it will be 1.

From Equation (30), we know that this Markov chain is a finite irreducible chain. The synchronization state is an absorptive state, and has global reachability, producing Equation (32):

(32) $$P^{n}=\left[\begin{array}{cccc} 1 & 0 & \cdots & 0\\ \vdots & \vdots & & \vdots \\ 1 & 0 & \cdots & 0 \end{array}\right],when\;n\geq \frac{T}{2\Delta t}$$

The two-node system reaches a steady state after *T*/2Δ*t* steps starting from any initial state. Because the steady state is an absorptive state, the algorithm is converged and stable.

### 4.2. Stability of the Multi-Node Network

The stability of the two-node network can be extended to verify the stability of a multi-node network. In a multi-node network, a specific node can be selected as a reference node, and the corresponding discrete phase differences of the other nodes can be calculated. These phase differences constitute a vector, and all the possible vectors constitute a state space of the multi-node system. The process of the algorithm is transformed into the state transition process of the Markov chain.

According to Equation (31), for any two adjacent nodes, the steady state is an absorbing state and has global reachability while the initial state is a non-absorbing state. As a multi-node network is composed of multiple pairs of two-node networks, when all nodes stochastically match each other, the steady state of the state space also has global reachability [31]. Through deeply connecting and limiting the number of nodes in the network, the composition of the Markov chain is a finite irreducible chain. Therefore, the convergence of the continuous-time Markov process in [31] is also applicable to the discrete-phase Markov chain model of the network. First, the state space in a Markov chain is represented by a set of state vectors ***S*** = {***s***_1_,***s***_2_,…,}. Each element in *S* represents a possible state vector of the system. Suppose the steady state in the Markov chain constitutes the set *C*. The remaining states are non-persistent states, constituting a set *N*. *P* represents the Markov chain transition probability matrix, and Equation (33) can be deduced:

(33) $$P^{l}=(p_{xy}^{l})$$

Then:

(34) $$\sum_{y\in C\cup N}p_{xy}^{\left(l\right)}=1$$

(35) $$\underset{l\to \infty }{\lim}\sum_{y\in C\cup N}p_{xy}^{\left(l\right)}=1$$

(36) $$\underset{l\to \infty }{\lim}\sum_{y\in N}p_{xy}^{\left(l\right)}+\underset{l\to \infty }{\lim}\sum_{y\in C}p_{xy}^{\left(l\right)}=1$$

For ∀*x,y* ∈ ***N***, because *N* is a non-persistent state set:

(37) $$\underset{l\to \infty }{\lim}\sum_{y\in N}p_{xy}^{\left(l\right)}=0$$

According to the theory of Markov chains, we know that there always exists a value of transfer times *n* that can realize ***P****^n^* > 0 and ***P****^n=^*^1^***p***^T^, ***p*** ∈ ***C***. According to Equation (36), since the steady state is the absorptive state, as the algorithm iterates, other nodes tend to be synchronized and finally reach stability.

## 5. Simulation Verification

In this section, we verify the stability of the synchronization algorithm proposed in Section 3 using Matlab. We divide the simulations into two groups. One group employs the typical RFA mechanism based on the M&S model and the other one employs the target algorithm to perform synchronization experiments in a non-fully connected mesh network. By recording the phase information of the nodes during the process, the influence of different algorithms on the synchronization can be analyzed.

### 5.1. Simulation Parameters

**Figure 5:**
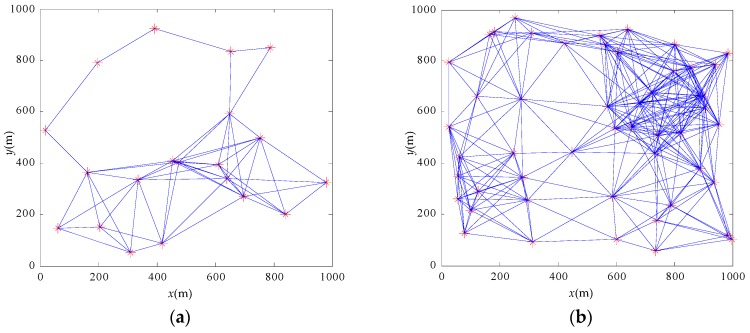
Layout and topology of the network. (**a**) The 20-node network; (**b**) the 50-node network.

In order to enhance the contrast between the algorithms, there are several common simulation parameters in both groups. In the simulation experiment, 30 or 50 nodes are randomly arranged in a 1000 m × 1000 m area as shown in Figure 5. The communication distance of the node is 350 m, and the simulation time is 300 s with a 4 μs simulation step. The initial phase of the nodes is randomly distributed between 0 and *T*. The period of the cycle is 1.048576 s. The refractory period of the node is set to 16 μs. The communication delay and frequency drift of the node both influence the synchronization performance [11]. To simulate an extremely poor communication environment in spite of the compensation, we set a 50 ppm frequency drift and 100 μs communication delay. Additionally, a packet loss rate up to 20% is set which is uniformly distributed.

The state vector of each node is recorded at intervals of 1 s and the phase difference of any two points in the network can be calculated according to Equation (3). The maximum value of the difference is taken as the synchronization error. In the typical RFA synchronization mechanism, the coupling coefficient is set as 0.5, 0.01, and 0.005. In the multiscale phase dynamics, the number of multiscale layers is set to three, and the quantization levels are 64, 32, and 32. The minimum quantization resolution is 16 μs and the coupling meets Equation (16) while the coupling coefficient is calculated by Equation (15) dynamically.

### 5.2. Simulation Results

#### 5.2.1. Contrast Test with 20 Nodes

**Figure 6:**
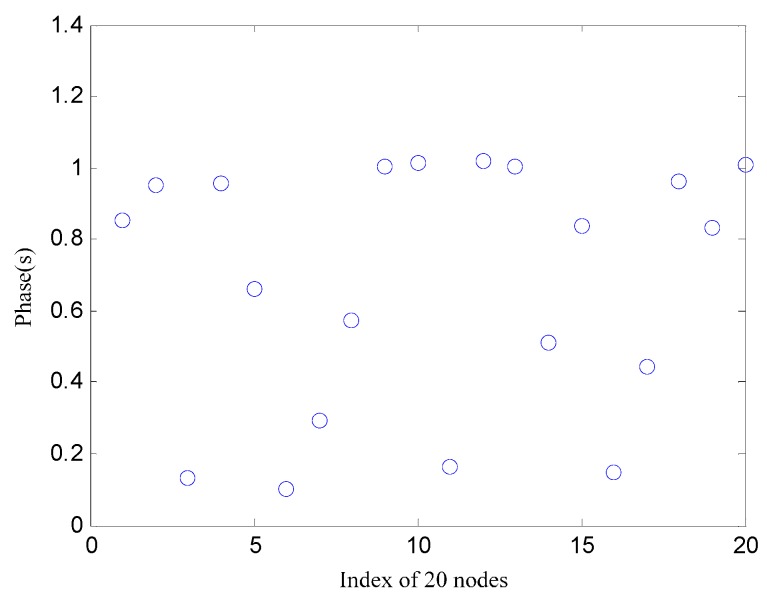
The 20 nodes in non-synchronized states.

**Figure 7:**
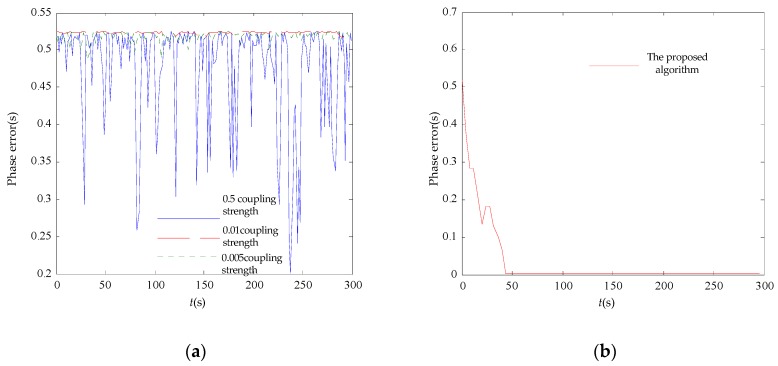
20-node synchronization error comparison. (**a**) RFA synchronicity error; (**b**) the proposed algorithm synchronicity error.

Figure 6 shows the initial phases where 20 nodes are in the non-synchronized state. The synchronization error in the 20-node network is shown in Figure 7. It can be seen that the initial phase of the node set is dispersed in both algorithms. Only the multiscale phase dynamics stimulation achieves phase convergence, and the convergence time is 44 s with an 80 μs error while the standard deviation well is under 26.14 μs. The multiscale phase dynamics algorithm is superior to the RFA in terms of the stability of the algorithm.

#### 5.2.2. Contrast Test with 50 Nodes

**Figure 8:**
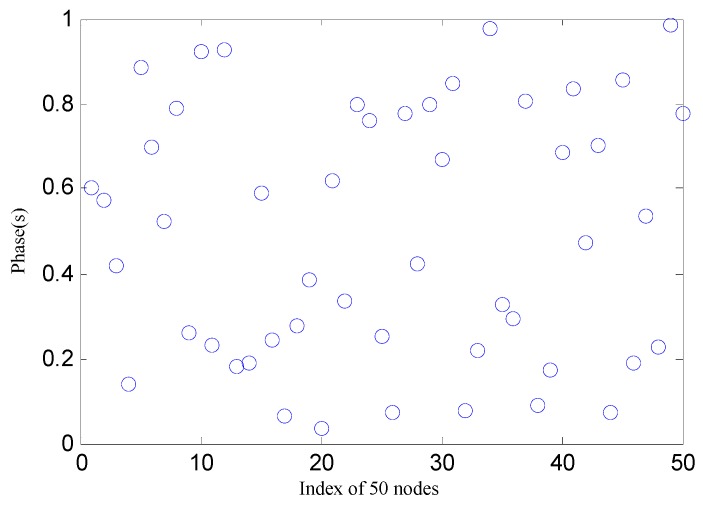
The 50 nodes in non-synchronized states.

**Figure 9:**
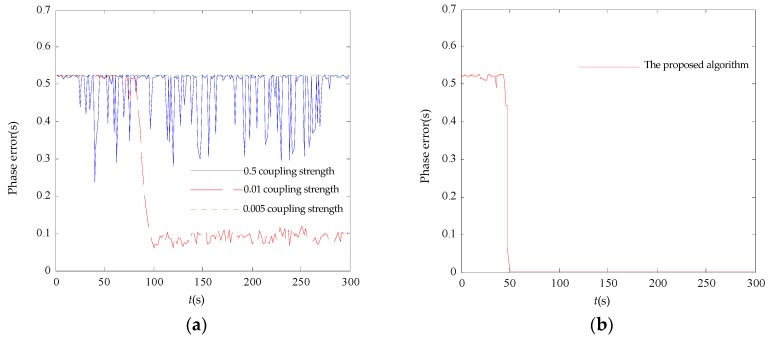
50-node synchronization error comparison. (**a**) RFA synchronicity error; (**b**) the proposed algorithm synchronicity error.

Figure 8 shows the initial phase where 50 nodes are in the non-synchronized state. The synchronization error in the 50-node network is shown in Figure 9. We can see that the initial phase of the node set is dispersed in both algorithms. The RFA is only able to achieve phase convergence with a coupling strength of 0.01, and the target algorithm achieves phase convergence, while the rest are unstable. The RFA converges in a spread interval of 0.11 s while the target algorithm converges in a spread interval of 0.032 ms with the standard deviation well under 0.00876 ms. These results indicate that the synchronization algorithm based on the proposed mechanism is superior to the traditional RFA in both convergence speed and precision.

### 5.3. Results Analysis and Discussion

#### 5.3.1. Comparison with RFA

The simulation results show that the target algorithm is stable in both networks, while the RFA is stable only in the 50-node network. Additionally, the target algorithm is 50% faster and much more accurate. In terms of synchronization performance, the RFA is difficult to stabilize in the 20-node network because of low network connectivity and needs an appropriate coupling coefficient for convergence in the 50-node network. In contrast, the target algorithm is stable and has a better performance under all conditions. The proposed algorithm is proven to have high stability, high convergence speed, and high synchronization precision compared with RFA. Additionally, the target algorithm has similar advantages as the firefly-inspired algorithm.

#### 5.3.2. Comparison with Traditional Synchronization Protocols

The target algorithm represents a radically different method. Compared with the traditional synchronization protocols, all of the nodes behave equally in our target algorithm. Unlike the traditional synchronization protocols, there are no root nor reference nodes. Our target algorithm is not affected by the network layer. As a result, it is more robust to the single points of failure and the flexible links, which is one of the main attractions. In addition, it is more conducive to network expansion. Nevertheless, comparing with traditional synchronization protocols, there is still a certain gap in terms of accuracy. For instance, as shown in Section 5.2, the target algorithm achieves 32 μs which is better than RFA, but still slightly less than the reported 14 μs accuracy of Flooding Time Synchronization Protocol (FTSP) [9]. We believe that this accuracy can be increased by adjusting the quantization layer and time resolution in each layer and much work remains to be done. Currently, the accuracy of our target algorithm is sufficient for sleep management and time-division multiplexing.

#### 5.3.3. Discussion on Power Consumption

Although the algorithm does not achieve significant improvement in energy consumption, it is still more energy efficient than RFA because of its higher convergence speed. However, as described in Section 2.1.2, each synchronization packet cannot correct the local clock completely but by a little step. Therefore, comparing with the traditional synchronization protocols, more synchronization packets and more time will be needed for reaching the convergence, which means more power consumption. Once the time synchronization is achieved, the synchronization mainly depends on the internal accuracy of the components or clock drifts of the clock system given by the manufacturing process. The traditional synchronization protocols are adaptive. Thus, in this case, they can schedule the synchronization packets on demand. Similarly, if the clock drift is not too large, our target algorithm can reduce the energy consumption by operating at a much lower transmission frequency and combining the synchronization packet with other network transmission packets. The transmission frequency depends on the amount of out-of-phase synchronization packets received during a period of time. So it will be competitive to the traditional synchronization protocols during the maintaining period in terms of the energy consumption. In the future work, we consider the algorithm optimization as follows: When join in the network, each node will initialize its phase by traditional synchronization algorithm, and use our algorithm to tighten the accuracy and maintain synchronicity. It will greatly speed up the synchronization and reduce the energy consumption before convergence.

## 6. Algorithm Verification on as Hardware Platform

### 6.1. Hardware and Experiment Design

In order to verify the practicality and validity of the synchronization algorithm, a high-precision synchronization acquisition platform is designed with 30 wireless nodes, an acquisition board, and an industrial computer. The algorithm is implemented on the MCUs (JN5148) in wireless nodes [33]. The phase synchronization emerges from the interaction of the nodes by wireless communication, while the phase value is acquired by a wired National Instruments (NI) synchronization acquisition platform. The unified time reference is provided by the synchronous acquisition to verify the effectiveness of the algorithm.

**Figure 10:**
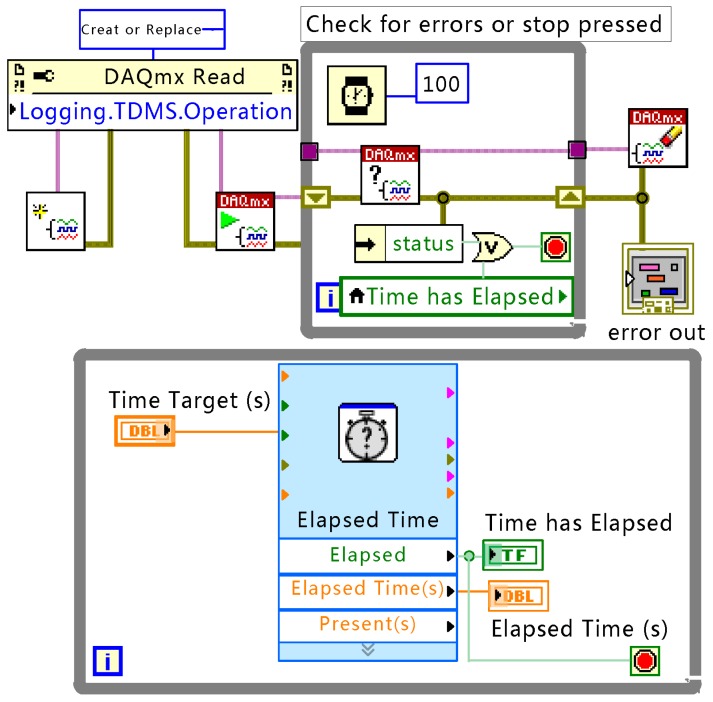
The block diagram of the acquisition program.

In this experiment, the integrating dynamics are calculated by the timer on the MAC layer in the RF module, and the synchronization period is 1.0486 s. The minimum quantization resolution is 16 μs. In order to maintain a certain degree of consistency with the simulation, as in the simulation, the number of multiscale layers is three, and the quantization levels are 64, 32, and 32. The refractory period is set to 50 μs. To form a non-fully connected network in the laboratory, it is necessary to restrict each node to have at most eight neighbor nodes with which they can communicate directly, and this limits the network connectivity up to a factor of four. Meanwhile, a packet loss rate up to 20% is set by the software. When a node finishes a synchronization period, its MCU will pull up the level of a GPIO port for 300 ms. The GPIO is sampled by NI’s PXIe6358 30-channel digital signal acquisition [34], and the sampling frequency is 100 ksps. For visual observation, the IO port is connected to an LED. The GPIO ports are connected via wires to the digital input channel of the acquisition card while the synchronization is implemented wirelessly. Labview is employed to acquire the real phase value of the 30 nodes on the industrial computer for verification. The block diagram of the acquisition program (the Virtual Instrument block diagram in Labview) is shown as Figure 10.

### 6.2. Experimental Results and Discussion

**Figure 11:**
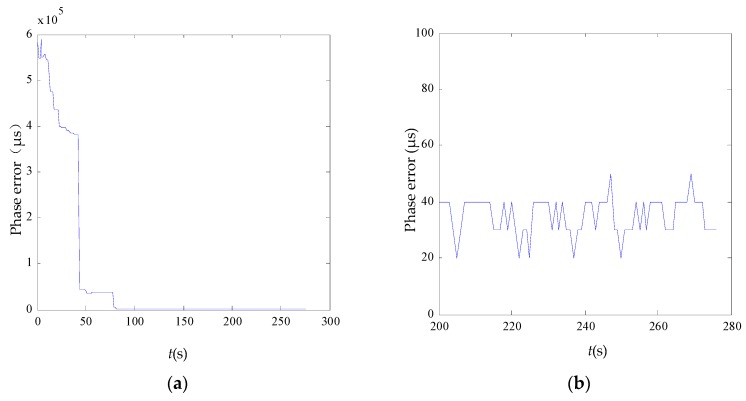
The synchronization error (**a**) during the whole process and (**b**) from 200 to 280 s.

**Figure 12:**
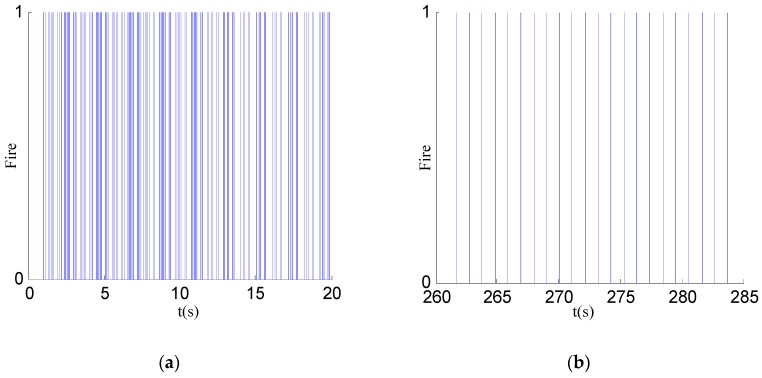
Temporal dynamics of 30 nodes. (**a**) Unsynchronized state; (**b**) synchronized state.

**Figure 13:**
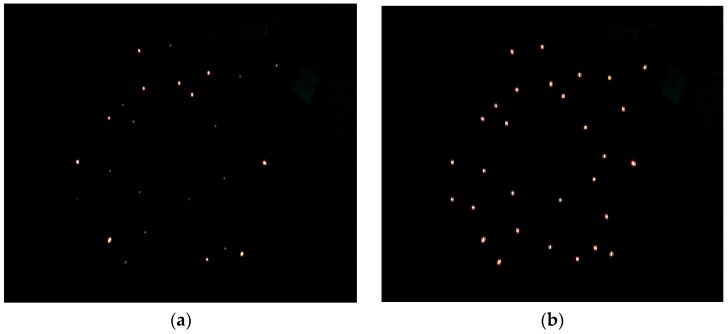
Node synchronization effect. (**a**) Unsynchronized state; (**b**) synchronized state.

It can be seen in Figure 11 that each node achieves phase synchronization. The maximum error spread of synchronization at each sampling point in time is plotted in Figure 11a. Due to the multiscale phase mechanism, synchronization of the node is divided into three phases. The maximum quantization resolution leads to early rapid decrease of the synchronization error, and the minimum quantization resolution reduces the jitter at later times. Figure 11b shows that the final error spread is in a 50 μs interval with the standard deviation well under 15.42 μs. And the average oscillation period is 1.0485904 s. However, interferences in network may lead to a synchronization error jitter after synchronization, but due to the synchronization algorithm, can still be limited to a certain range. Figure 12 shows the temporal dynamics of 30 nodes in an unsynchronized and synchronized state. In this figure, a vertical bar represents a fire occurrence. Figure 12a shows the chaotic fires in the unsynchronized network. By comparison, Figure 12b has the neat and regular fires in a synchronized state. Figure 13 shows the node synchronization effect. In Figure 13a, the network is in an unsynchronized state. The state at 50 s is shown in Figure 13b, and it can be seen that the network has reached synchronization which requires less than 1600 packets. Therefore, the algorithm achieves synchronization with stability and high performance. The effectiveness of the discrete phase dynamics and algorithm were proven to have a high practical value.

## 7. Conclusions and Extensions

In this paper, discrete phase dynamics at multiscale quantitative levels is proposed. The stochastic coupling algorithm is employed to achieve synchronization in a WSN with a complex topology. The stability of the algorithm is verified in this paper. The simulation results show that the proposed algorithm is more stable in both a 20-node and 50-node network, while RFA is stable only in a 50-node network with a coupling strength of 0.01. Finally, the target algorithm is implemented in a realistic WSN with a complex topology for verification. The performance of the proposed algorithm is only demonstrated preliminarily. It is necessary to conduct further studies to determine the effect of the parameters in the proposed algorithm on the performance and the energy consumption will be taken into account. Because this algorithm is a software-based algorithm, it can and will be transplanted and tested on the ATmega256RFR2 chip for supporting sleep management functions. Nevertheless, the proposed algorithm improves the stability of the firefly synchronization algorithm and reduces the design difficulty of firefly-inspired synchronization algorithms for WSNs with complex topologies.
